# Effective Cardiac Myocyte Differentiation of Human Induced Pluripotent Stem Cells Requires VEGF

**DOI:** 10.1371/journal.pone.0053764

**Published:** 2013-01-10

**Authors:** Lei Ye, Sophia Zhang, Lucas Greder, James Dutton, Susan A. Keirstead, Mike Lepley, Liying Zhang, Dan Kaufman, Jianyi Zhang

**Affiliations:** 1 Division of Cardiology, Department of Medicine, University of Minnesota Medical School, Minneapolis, Minnesota, United States of America; 2 Stem Cell Institute, University of Minnesota Medical School, Minneapolis, Minnesota, United States of America; 3 Department of Genetics, Cell Biology, and Development, University of Minnesota, Minneapolis, Minnesota, United States of America; 4 Department of Integrative Biology and Physiology, University of Minnesota, Minneapolis, Minnesota, United States of America; 5 Department of Biomedical Engineering, University of Minnesota, Minneapolis, Minnesota, United States of America; Northwestern University, United States of America

## Abstract

Perhaps one of the most significant achievements in modern science is the discovery of human induced pluripotent stem cells (hiPSCs), which have paved the way for regeneration therapy using patients’ own cells. Cardiomyocytes differentiated from hiPSCs (hiPSC-CMs) could be used for modelling patients with heart failure, for testing new drugs, and for cellular therapy in the future. However, the present cardiomyocyte differentiation protocols exhibit variable differentiation efficiency across different hiPSC lines, which inhibit the application of this technology significantly. Here, we demonstrate a novel myocyte differentiation protocol that can yield a significant, high percentage of cardiac myocyte differentiation (>85%) in 2 hiPSC lines, which makes the fabrication of a human cardiac muscle patch possible. The established hiPSCs cell lines being examined include the transgene integrated UCBiPS7 derived from cord blood cells and non-integrated PCBC16iPS from skin fibroblasts. The results indicate that hiPSC-CMs derived from established hiPSC lines respond to adrenergic or acetylcholine stimulation and beat regularly for greater than 60 days. This data also demonstrates that this novel differentiation protocol can efficiently generate hiPSC-CMs from iPSC lines that are derived not only from fibroblasts, but also from blood mononuclear cells.

## Introduction

Following transmural myocardial infarction (MI), left ventricular (LV) remodelling with chamber dilation and hypertrophy occurs to compensate for the loss of contracting myocardium. Although stable LV remodelling may be achieved for a period of time, progressive myocardial dysfunction can develop and ultimately lead to overt congestive heart failure (CHF). The mechanisms that contribute to the transition from the compensated state to CHF remain unclear, but may be related to progressive contractile dysfunction and over-stretching in the region of viable myocardium that surrounds the infarct (border zone, BZ). [1], [2].

Currently, the available therapeutic options for heart failure due to transmural LV infarct are limited. Although it is a consistent observation in literature that cell transplantation improves LV contractile function, the cell engraftment rate and regeneration of cardiac myocytes is usually very low [3]–[10]. Perhaps one of the most significant achievements in modern science was the discovery of human induced pluripotent stem cells (hiPSCs) [11], [12]. Induced pluripotent stem cells (iPSCs), cells that can differentiate into all cell types including cardiac myocytes, are a type of pluripotent stem cell derived from adult somatic cells that have been genetically reprogrammed back to an embryonic stem cell-like state through the forced expression of genes and factors important for maintaining the defining properties of embryonic stem cells (ESCs) [13]–[16]. Shinya Yamanaka and his coworkers were able to show that mouse embryonic fibroblasts (MEFs) could be converted into germline-competent induced pluripotent stem cells by retroviral expression of four transcription factors: OCT4, SOX2, KLF4 and c-MYC [13], [17]. This reactivation leads to a resetting of the epigenetic profile of a terminally differentiated somatic cell and activates the molecular circuitry of pluripotency [18], [19]. Cardiomyocytes derived from hiPSCs (hiPSC-CMs) have the potential for autologous cardiomyocyte transplantation therapy. hiPSC-CMs provide a better alternative to human embryonic stem cells (hESCs) derived cardiomyocytes (hESC-CMs) for two reasons. Firstly, hiPSC-CMs bypass political and ethical problems of damaging the human embryos; secondly, a large quantity of patient specific cardiomyocytes could be derived from hiPSCs thus allowing the use of autologous cardiomyocytes for transplantation. However, the methods to derive cardiomyocytes from hiPSC are limited and are iPSC lines specific. The origin of the donor somatic cells from the tissue, as well as the reprogramming method applied may have effects on the efficiency of cardiac myocyte differentiation.

Although it has been shown that cardiac progenitors transplanted into hearts with MI can repair the damaged myocardium at some level, there could be additional benefit in applying a prefabricated bioartificial cardiac tissue, a “cardiac muscle patch”, over the surface of a myocardial infarct to prevent scar expansion and overstretching of BZ myocytes. A “cardiac muscle patch”, formed by entrapping human cardiac myocytes in a poly-ethylene-glycolated (PEGylated) fibrin 3D porous biomaterial, has recently become possible [20], [21] as the basis for a bioartificial “cardiac muscle patch”. We have recently established novel 3D porous PEGylated fibrin biomaterial that can covalently bind to growth factors to create an optimal microenvironment for cells to reside [21]–[23] and differentiate. The PEGylated biomaterial also functions as a controlled, prolonged release of growth factors to mobilize endogenous cardiac progenitors and to prevent apoptosis [23], [24]. Our long term objective of this research is to develop a contracting human cardiac muscle patch that be transplanted on the surface of LV scar to effectively prevent LV bulging, reduce the LV dilatation and myocyte over-stretch, consequently prevent heart failure to occur. In the present project, we hypothesized that the novel cell differentiation protocol with the addition of VEGF can generate significant high percentage of cardiac myocyte differentiation. Here, we describe an effective method that has successfully differentiated cardiac myocytes from four established hiPSCs lines, UCBiPS7, BC-1, DriPS16, and PCBC16iPS, into hiPSC-CMs. UCBiPS7 was reprogrammed from human cord mononuclear blood cells using lentivirus, BC-1 was reprogrammed from human bone marrow CD34+ cells using non-integrate pEB-C5 vectors, DriPS16 was reprogrammed from neonatal human dermal fibroblast using lentivirus, and PCBC16iPS was reprogrammed from neonatal human dermal fibroblast using the non-integrating Sendai virus. In the current study, we report the differentiation protocol and efficacy in UCBiPS7 and PCBC16iPS. This differentiation protocol is unique in that it not only efficiently differentiates human cord blood cells derived from hiPSCs, which is an integrated hiPSCs, but it also efficiently differentiates the non-integrated hiPSCs, PCBC16iPS.

## Materials and Methods

### Culture of hiPSCs

Two hiPSC lines were investigated in the study: PCBC16iPS and UCBiPS7. Both cell lines used the same reprogramming factors: OCT4, SOX2, KLF4, and C-MYC. CBiPS7 was reprogrammed from human umbilical cord mononuclear blood cells (from cord blood bank, IBC code number: 0802E26309) using Lentivirus (IBC code Number: 1109H04828), while PCBC16iPS was reprogrammed from neonatal human dermal fibroblasts (ATCC, USA) using the non-integrating Sendai virus (IBC code number: 0804H31741). Both cell lines were cultured with mouse embryonic fibroblasts (MEFs) and regularly passaged every 6–7 days. Cells were cultured in hiPSC growth medium: 85% DMEM/F12 supplemented with 15% knockout serum, 8 ng/mL bFGF, 0.5× Penicillin-Streptomycin, 1× Non-essential amino acid (NEAA), 1 mM glutamine, and 55 µM mercaptoethanol.

### Differentiation hiPSCs into Cardiomyocytes

Four days before initiating differentiation, hiPSCs were dissociated into single cells and cultured as a monolayer in mTeSR1 medium on growth factor reduced Matrigel (BD, USA) coated plate. mTeSR1 medium was changed daily. When cells reached confluence, they were considered ready for differentiation. Cells were cultured with RPMI1640 medium (Life Technologies, USA) supplemented with B27 without insulin (Life Technologies, USA), 50 ng/mL Activin A (R&D systems, USA), and 25 ng/ml BMP-4 (R&D systems, USA) for 24 hours. On the next day, differentiation medium was replaced with RPMI1640 medium supplemented with B27 without insulin, 5 or 10 ng/mL VEGF (R&D systems, USA) for 72 hours. Then cells were cultured in RPMI1640 medium supplemented with B27 complete (Life Technologies, USA) and changed every 2–3 days. Generally, some cells started contracting on day-10 or 11 after initiating differentiation.

### Quantitative RT-PCR (QRT-PCR) Analysis

Undifferentiated hiPSCs, differentiating hiPSCs on day-1 after culture with Activin-A and BMP-4 for 24 hours (hiPSC-AB) and day-4 after culture with VEGF (hiPSC-ABV) for 72 hours, and cardiomyocytes on day-30 after initiating differentiation (hiPSC-CMs) were collected to quantify gene expression. The primers are listed in Table-1. GAPDH was used as an internal control. Gene expression level was calculated as relative to GAPDH expression. The isolation of total RNA and cDNA synthesis was carried out as described earlier [25]. The QPCR thermal cycling program for 40 cycles was: 1 cycle of enzyme activation at 95°C for 15 minutes, denaturation at 95°C for 30 seconds, annealing at 58°C for 30 seconds and extension at 72°C for 30 seconds.

**Table 1:** QRT-PCR Primers.

Gene Name	Gene Number	Sense	Anti-sense	Size
GAPDH	NM_002046	TCGACAGTCAGCCGCATCTTCTTT	ACCAAATCCGTTGACTCCGACCTT	94 bp
Oct3/4	NM_001173531	ATGCATTCAAACTGAGGTGCCTGC	AACTTCACCTTCCCTCCAACCAGT	192 bp
Sox2	NM_003106	CACATGAAGGAGCACCCGGATTAT	GTTCATGTGCGCGTAACTGTCCAT	192 bp
Nanog	NM_024865	CCCAAAGGCAAACAACCCACTTCT	AGCTGGGTGGAAGAGAACACAGTT	107 bp
				
Brachyury	NM_003181	AAAGAGATGATGGAGGAACCCGGA	AGGATGAGGATTTGCAGGTGGACA	108 bp
				
KDR	NM_002253	AGTGGCTAAGGGCATGGAGTTCTT	GGGCCAAGCCAAAGTCACAGATTT	119 bp
PDGFRα	NM_006206	CATGCCTGATGAAAGCTTTGGCGA	AGGATCCAGGCTAATGCACATCCA	189 bp
				
Nkx2.5	NM_001166175	TTAAGTCACCGTCTGTCTCCCTCA	ACCGACACGTCTCACTCAGCATTT	124 bp
Gata-4	NM_002052	ACCTGGGACTTGGAGGATAGCAAA	TCCCATCAGCGTGTAAAGGCATCT	169 bp
cTnI	NM_000363	TGACCTTCGAGGCAAGTTTAAGCG	TGTCCTCCTTCTTCACCTGCTTGA	143 bp
cTnT	NM_000364	TGCAGGAGAAGTTCAAGCAGCAGA	AGCGAGGAGCAGATCTTTGGTGAA	155 bp

### Immunohistochemistry Study

Undifferentiated hiPSCs were cultured on chamber slides with MEF feeders and immunostained for Oct3/4 and SSEA-4 expressions. Cardiomyocytes were microdissected from the contracting area and cultured for 5–6 days. Cells were fixed with 4% paraformaldehyde for 20 minutes at room temperature and permeabilized in 0.1% Triton X-100 at 4°C for 10 min. Cells were blocked with UltraV block (Thermo Scientific, USA) for 7 min. Primary antibodies, including monoclonal anti-Oct3/4 (Santa Cruz Biotechnology, USA), goat anti-SSEA-4 (Santa Cruz Biotechnology, USA), mouse anti- cTnT (Thermo Scientific, USA), rabbit anti-cTnI (Abcam, USA), goat anti-GATA-4 (Santa Cruz Biotechnology, USA), goat anti-Nkx2.5 (Santa Cruz Biotechnology, USA), rabbit anti-MLC2v (Proteintech Group, USA), and mouse anti-α sarcomere actin (α-SA, Sigma Aldrich, USA) in concentrations ranging from 1:50–1:100 were added to UltraV block buffer and incubated overnight at 4°C. On the second day, cells were incubated with UltraV block containing secondary antibodies for 1 hour at room temperature and followed by DAPI incubation. After a thorough wash, the cells were examined with a fluorescence microscope (Olympus, Japan).

### Cytofluorimetry of Differentiated hiPSCs for Cardiac Troponin T (cTnT) Expression

Differentiated hiPSCs on day-30 after initiating differentiation were trypsinized and re-suspended as single cells in glass tubes. Cells were fixed with 4% paraformaldehyde for 20 minutes at room temperature and permeabilized in 0.25% Triton X-100 at 4°C for 10 min. After incubation with UltraV block for 7 minutes at room temperature, mouse anti-cTnT or isotype control primary antibodies were added into designated tubes. After overnight incubation at 4°C, cells in designated tubes were washed and incubated with 1∶200 donkey anti-mouse IgG-FITC for 1 hour at room temperature for detection of cTnT. After thorough washing with PBS, cells were re-suspended in 0.25 mL PBS. Samples were analyzed using a FACS Aria instrument (BD Biosciences, USA). Cells with an adequate size and granularity were accounted for in the statistical analysis [26]. The percentage of cTnT^+^ cells/total cells was calculated.

### Electrophysiology Methods

CM electrical activity was measured using whole cell current clamp with patch electrodes (2–5 MΩ) containing 140 mM KCl, 1 mM MgCl_2_, 1 mM CaCl_2_, 11 mM EGTA, 5 mM HEPES, 1 mM glutathione, 3 mM ATP-2K, 2 mM glucose, 0.5 mM GTP-Na (pH 7.2, KOH). Cells were continuously superfused with extracellular solution containing (mmol/L): 146 NaCl, 3 KCl, 10 HEPES, 2 CaCl_2_, 2 MgCl_2_, 1.25 NaH_2_PO_4_, 1 Na pyruvate, and 10 D-glucose (pH 7.4, NaOH) at room temperature. Junction potentials and electrode resistance were nulled and data were acquired at 10 kHz using a Multiclamp 700 A amplifier and pClamp 9.2 software (Molecular Devices, Sunnyvale CA). Data were filtered off-line using a low pass Gaussian filter with a cut-off frequency of 2 kHz and plots were made using Prizm 6.2 (GraphPad Software Inc., San Diego, CA). Norepinephrine (100 µM) and carbachol (10 µM) were bath applied in the superfusate for 30–90 seconds using a valve operated perfusion system (VC-6; Warner Instrument, LLC, Hamden, CT, USA).

### Imaging Methods

Cells were loaded with 10 µM Fluo-3 AM (Life Technologies, Grand Island, NY, USA) dissolved in culture medium at 37°C for 30–90 minutes. Cells were superfused at room temperature with extracellular solution for at least 20 minutes to permit de-esterification before imaging. Fluorescence images were acquired using an Olympus BX-51W microscope outfitted with a Lambda DG-4 fluorescence light source and filter changer (Sutter Instrument, Novato, CA, USA) and a Paultek intensified charge-coupled device. The excitation filter was 470 nm/40 nm and the emission filter was 525 nm/50 nm. Metamorph/Metafluor 6.2 Imaging Suite (Molecular Devices, Sunnyvale, CA, USA) was used to acquire images at 200 msec intervals. Images were background subtracted and displayed in a false color scale.

### Statistics

Statistical analysis was performed using SPSS (version 20.0). All data is presented as mean± standard error means (SEM).

## Results

### Characterization of hiPSCs

**Figure 1:**
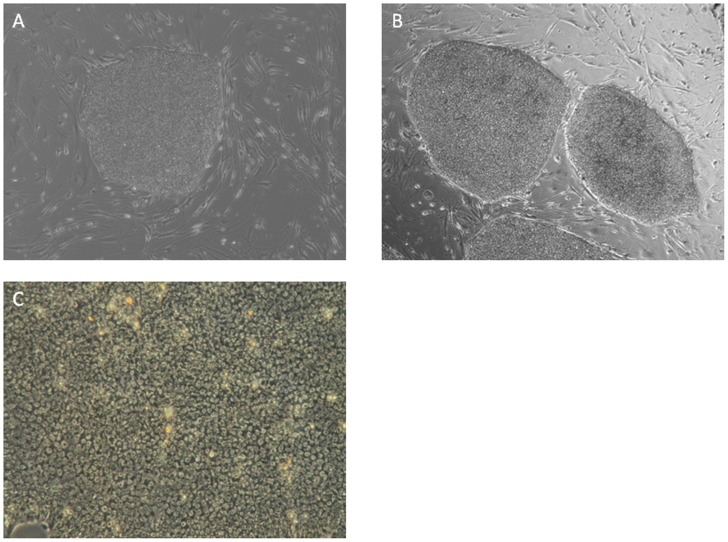
Morphological characteristics of hiPSCs. Typical phase contrast pictures of UCBiP7 (A) and PCBC16ShiP (B) grew in flat and compact colonies with distinct cell borders in monolayer culture with irradiated MEF (Magnification = 40x). (C) Microscopic 100× view of a colony of undifferentiated hiPSC: high nucleus-to-cytoplasm ratios, and prominent nucleoli.

**Figure 2:**
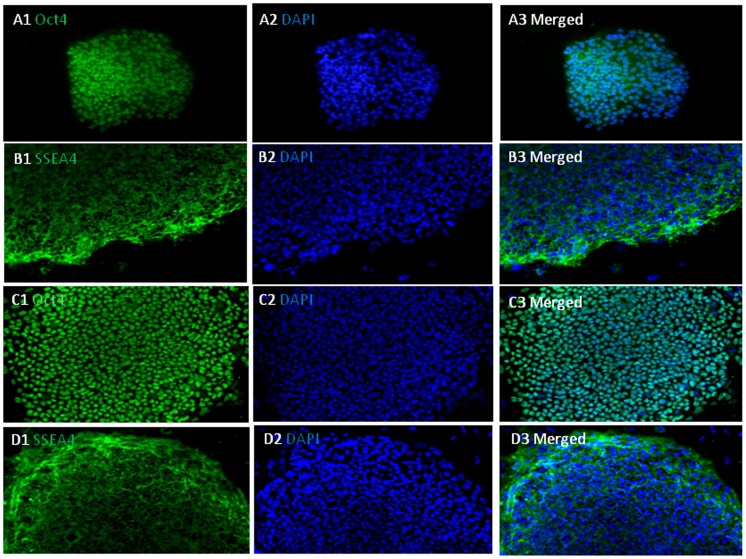
Pluripotent stem cell markers of UCBiP7 and PCBC16iPS. UCBiP7 immunostained for Oct3/4 (A1–3) and SSEA-4 (B1–3) protein expressions. PCBC16iPS immunostained for Oct3/4 (C1–3) and SSEA-4 (D1–3) protein expressions. (Magnification = 100x).

Both UCBiPS7 and PCBC16iPS cell lines had typical morphological characteristics of hiPSCs (Figure 1). They grew in flat and compact colonies with a distinct border in monolayer culture with irradiated MEF. They had high nucleus-to-cytoplasm ratios and prominent nucleoli. They expressed pluripotent stem cell markers: Oct4 and SSEA-4 (Figure 2). These observations suggest that both UCBiPS7 and PCBC16iPS cell lines have typical characteristics of hESCs.

### Cardiac Myocyte Differentiation of hiPSCs

Generally, hiPSC-derived cells started contracting on day-10 or 11 after initiating differentiation. Isolated contracting areas were found on the first few days after initiating differentiation and expanded to form a large contracting area. Online Movie S1, S2, and S3 show the contracting of UCBiPS7 on day-1, -5 and -10 after initiating contracting. Movie S4, S5, and S6 show the contacting of PCBC16iPS on day-2, -6 and -11 after initiating contracting.

**Figure 3:**
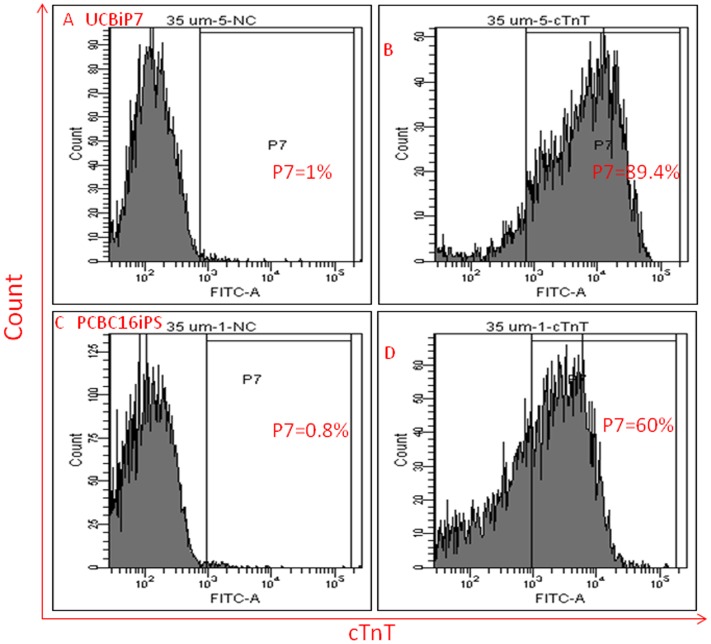
Flow cytometry analysis for differentiation efficiency based on cTnT protein expression. Isotype control of UCBiP7 (A) and PCBC16iPS (C) for cTnT protein expression. More than 88% of UCBiP7 (B) and 59% of PCBC16iPS differentiated cell expressed cTnT on day-30 after initiating differentiation.

FACS analysis demonstrated that 85.4±7% of a whole differentiated cell population of UCBiP7 was cTnT^+^ cells on day-30 after initiating differentiation. The highest differentiation efficiency reached was 88% (Figure 3). The overall differentiation efficiency was 54±3% for PCBC16iPS and the highest differentiation efficiency was 59%. These suggest that the current protocol has a higher differentiation efficiency in cord blood derived UCBiP7 than dermal skin derived PCBC16iPS.

Fluorescence immunostaining showed that 1–2% of all differentiated cells expressed CD31 protein suggesting these cells might be endothelial lineage cells. However, this might be by-products of differentiated cardiomyocytes from hiPSCs.

### Gene Expression Profile

**Figure 4:**
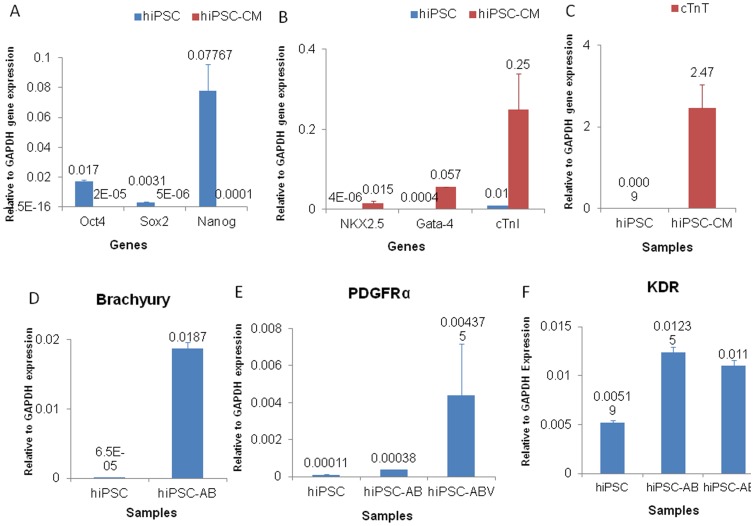
Gene expression profile of undifferentiated and differentiated hiPSCs. (A) Pluripotent stem cell markers of undifferentiated hiPSCs and differentiated hiPSC-CM. Gene expression levels of cardiomyocyte specific transcription factors and myofilament protein (B&C). Undifferentiated and differentiated hiPSCs for Brachyury (D), PDGFR-α (E), and KDR (F) gene expressions. (hiPSC-AB: hiPSCs treated with activin-A and BMP-4; hiPSC-ABV: hiPSCs treated with activin-A, BMP-4 and VEGF).

The gene expression levels of pluripotent stem cell markers, Oct4, Sox2, and Nanog, were significantly down-regulated after differentiation into cardiomyocytes (Figure 4A). The gene expression level of transcription factors for cardiac myocyte differentiation, including Nkx2.5 and GATA-4, were significantly up-regulated in differentiated cells (Figure 4B). Similarly, the gene expression levels of cardiomyocyte filament proteins, cardiac troponin I (cTnI) and cTnT, were also significantly up-regulated (Figure 4B&C). These suggest that the current differentiation protocol can successfully down-regulate the gene expression levels of pluripotent stem cell markers and up-regulate cardiomyocyte specific transcription factors and proteins. To investigate the differentiation pathway of the current protocol, brachyury, PDGFR-α, and KDR gene expression levels were determined. It was found that the synergy between Activin-A and BMP-4 significantly up-regulated brachyury (>250 fold), PDGFR-α (>3 fold), and KDR (>2 fold) gene expressions after 24 hours (Figure 4D–F). VEGF further increased PDGFR-α gene expression level, and maintained KDR expression level (Figure 4E&F).

### Characterization of hiPSC-CMs

**Figure 5:**
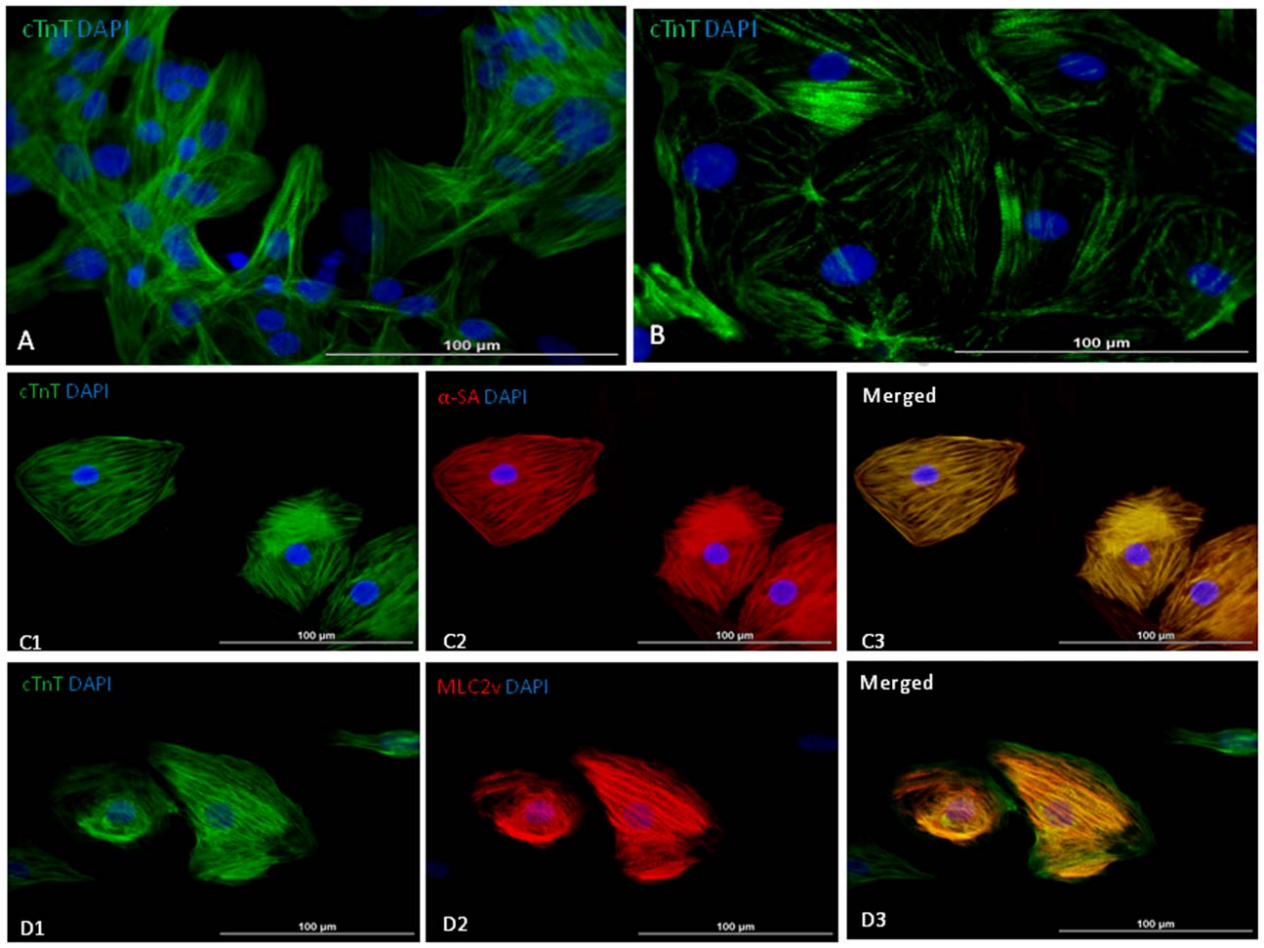
Characterization of hiPSC-CMs. Cardiomyocytes form UCBiP7 (A) and PCBC16iPS (B) expressed cardiomyocyte specific myofilament protein: cTnT. (C1–3) Striation formation of hiPSC-CM as evidenced by dual immunostaining for cTnT and α-sarcomere actin (α-SA) expressions (C1–3), or cTnT and myosin light chain 2v (MLC2v) expressions (D1–3). (Bar = 100 µm).

**Figure 6:**
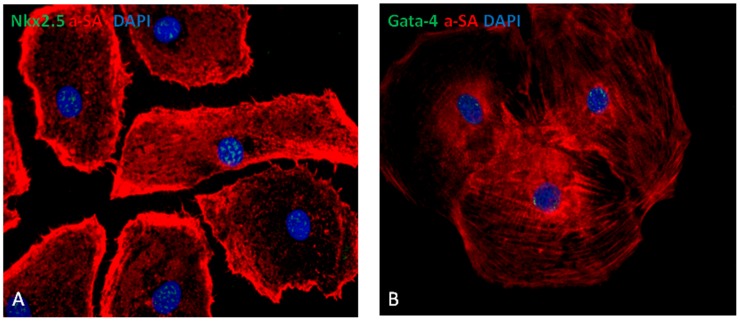
Cardiac transcription factor expression of hiPSC-CMs. (A) Nkx2.5 and (B) Gata-4 expressions in cardiomyocyte fluorescence immunostained for -sarcomere actin (α-SA). (Magnification = 400x).

To investigate the expression of cardiac transcription factors and myofilament protein, we micro-dissected and enzymatically isolated contracting cells on day-30 after initiating differentiation. Cells were used for immunohistochemistry to determine the myofilament protein expression. hiPSC-CMs differentiated from both cell lines expressed cTnT, a cardiac specific myofilament protein, and formed striations (Figure 5A&B). Dual staining showed that almost 100% hiPSC-CMs co-expressed α-sarcomere actin (α-SA) from both cell lines, and only 20–30% of cells co-expressed myosin light chain 2v isoform (MLC2v), a specific ventricular myosin light chain (Figure 5C&D). Immunostaining for cardiac transcription factor expression showed that Nkx2.5 and Gata-4 were expressed in nuclei of hiPSC-CMs (Figure 6). These studies demonstrate the expression of cardiac-specific transcription factors and myofilament proteins in differentiated hiPSC-CMs, which confirms their differentiation into cardiac myocytes.

### Electrophysiology Analysis

**Figure 7:**
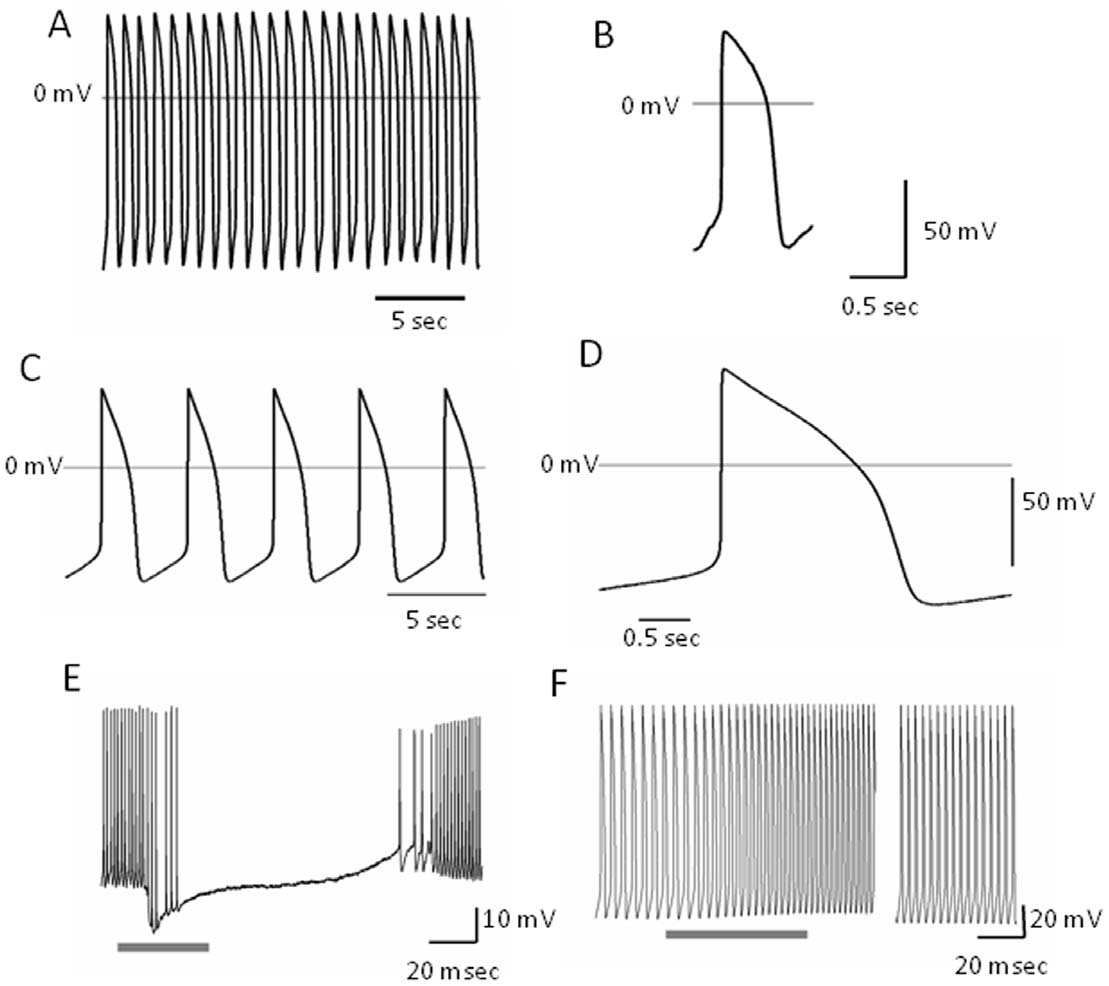
Electrophysiological properties of hiPSC-CMs. Representative whole cell recordings of atrial- (A&B) and ventricular-like (C&D) action potentials from hiPSC-CMs. Grey line indicates 0 mV. Right, single action potential at an expanded timescale taken from traces on the left. (E) 10 µM carbachol resulted in a decrease in spontaneous action potential frequency. (F) 100 µM norephinephrine resulted in an increase in spontaneous action potential frequency. Gap in horizontal axis –195 sec. Drug applications were during the times indicated by the gray bars at the bottom of traces in E and F.

hiPSC-CMs exhibited spontaneous cardiac-like action potentials. Action potential shape in some cells resembled ventricular-like action potentials with long repolarization phases and low frequency of spontaneous action potentials (Figure 7A&B), whereas others were more atrial-like action potentials, with shorter depolarization phases and higher frequency of action potentials (Figure 7C&D). These action potentials are similar those observed in other cardiomyocytes derived from hiPSC-CMs [27]. Bath application of the cholinergic agonist, carbachol (10 µM) resulted in a decrease in spontaneous action potential frequency (Figure 7E), while application of the adrenergic agonist, norephinephrine (100 µM), resulted in an increase in spontaneous action potential frequency (Figure 7F). Calcium imaging experiments revealed regular oscillations of intracellular calcium concentration that were often synchronized among cells within clusters (on-line movie S7). These data suggest that hiPSC-CMs are functionally coupled.

## Discussion

This study is the first to demonstrate that Activin-A/BMP-4/VEGF protocol efficiently differentiates cardiomyocytes from human umbilical cord blood mononuclear cell derived hiPSCs, UCBiP7. This protocol also works efficiently for non-integrated hiPSCs, PCBC16iPS.

The efficiency of cardiac differentiation from hiPSCs has significant variability depending on the cell origin and reprogramming method [28], [29]. Thus, a cell line-specific differentiation protocol may be preferred. Here, we describe a cardiomyocyte differentiation protocol that efficiently differentiates integrated (cord blood cells) and non-integrated (neonatal dermal skin fibroblasts) hiPSCs. The fact that the protocol works well with the very disparate hiPSC lines, demonstrates the potential of a universal differentiation protocol.

Zhang et al., [27] demonstrated that Matrigel in combination with Activin-A/bFGF/BMP-4 promotes cardiogenesis. They demonstrated that using Matrigel as an extracellular matrix (ECM) promoted an epithelial-to-mesenchymal transition and enabled robust cardiac differentiation when complemented by growth factor signaling. Three hiPSC lines (DF6-9-9T, DF19-9-7T and DF19-9-11T) derived from foreskin fibroblasts without integration of vector and transgene sequences and a lentiviral-generated hiPSC line IMR90 clone 4, reprogrammed from human lung fibroblasts, were studied. In the current study, the PCBC16iPS cell line is also a non-integrated hiPSCs derived from neonatal dermal skin fibrobalsts, while UCBiPS7 is an integrated cell line derived from cord blood cells. We tested the Matrix Sandwich Method for both cell lines. Though it successfully differentiated PCBC16iPS to contracting myocytes with high efficiency, it did not work for UCBiPS7 cell line. It is possible that cell origin of hiPSCs not only has significant impact on choice of reprogramming factors and reprogramming efficiency, but also affects differentiation efficiency. iPSCs may have memory of parental source and therefore have low differentiation efficiency into cells of other tissue types. Kim et al. [30] showed that iPSCs reprogrammed from peripheral blood cells could be efficiently differentiated into hematopoietic lineage cells. However, these cells showed very low differentiation efficiency into neural cells. Similarly, Bar-Nur et al., [31] demonstrated that iPSCs reprogrammed from human pancreatic islet β cells have an increased ability to differentiate into insulin-producing cells both in vitro and in vivo. These studies suggest that epigenetic memory will predispose iPSCs to differentiate more readily into the original cell type. Thus, it is possible that Matrigel in combination with Activin-A/bFGF/BMP-4 may work efficiently for cells originating from fibroblasts. However, this protocol may not be able to efficiently differentiate hiPSC originated from blood cells, such as UCBiPS7 based on the fact that the foreskin fibroblasts originate from ventral midline mesoderm whereas the blood cells come from aorta-gonad-mesonephros (AGM).

Besides cell origin, the differentiation process is critically dependent on the chemokines and growth factors, the time of addition, and the time of removal of growth factors. The current differentiation method combined Activin-A and BMP-4 for mesodermal induction, followed by VEGF treatment for cardiac mesodermal commitment. It is known that Activin-A alone induces mesoderm from hiPSC, while short-term BMP-4 treatment initiates mesoderm induction in human embryonic stem cells [32]. The synergy between Activin-A and BMP-4 aims to efficiently promote the initial EMT process leading to the generation of a population of mesodermal progenitors. 2:1 ratio of Activin-A/BMP-4 efficiently up-regulated brachyury gene expression by more than 250 fold, suggesting that this combination successfully induced mesoderm from hiPSCs within 24 hours. VEGF has been shown to promote KDR^+^ cardiovascular progenitor cell development from hESCs [33]. Thus, we chose VEGF to commit cells further to cardiac mesoderm within 3 days as evidenced by up-regulated KDR and PDGFRα expression. Yang et al., [33] combined Activin-A, BMP-4, and basic fibroblast growth factor (bFGF) for induction of mesoderm in 3 days, while VEGF and dickkopf homolog 1 (DKK1) for cardiac mesoderm commitment in 4 days. They demonstrated that a KDR^low^/c-KIT^neg^ population that can generate cardiomyocytes could be obtained using this protocol. The concentration of Activin-A (3–10 ng/mL) and BMP-4 (10 ng/mL) used are low in their study as compared with the current study. This may explain the less efficient induction of mesoderm within 24 hours using in their study.

We used VEGF alone for cardiac mesoderm commitment for 3 days. The gene expression of KDR and PDGFRα was significantly up-regulated. Kattman et al., [34] demonstrated that KDR+/PDGFRα+ population can generate highly enriched cardiomyocytes up to 80%. However, Activin-A, BMP-4, and VEGF were simultaneously used for induction of cardiac mesoderm in embryonic body (EB) of mouse iPSCs, while Activin-A, BMP-4, and bFGF were simultaneously used in EB of hiPSCs. We found that the combination of Activin-A and BMP-4 also increased the KDR and PDGFRα gene expression levels within 24 hours. VEGF further up-regulated PDGFRα gene expression level by 11 fold. These are partially consistent with Kattman’s finding that Activin-A and BMP-4 can bring about induction of cardiac mesoderm [34]. Combining these, VEGF promotes cardiomyocytes differentiation by activating Flk-1 by ERK [35] and PDGFRα [34].

Recently, Lian et al., [36] showed that sequential treatment of hiPSCs with glycogen synthase kinase 3 inhibitors followed by inducible expression of β-catenin shRNA or chemical inhibitors of Wnt signaling produced a high yield of virtually (up to 98%) pure functional human cardiomyocytes from three hiPSC lines, which were reprogrammed from human fibroblasts. This is the first study that demonstrates efficient and robust generation of cardiomyocytes from multiple hiPSC lines solely via small molecule modulation of regulatory elements of Wnt/β-catenin signaling. This provides a new differentiation strategy that efficiently differentiates hiPSCs into cardiomyocytes. However, its efficacy in hiPSCs derived from blood cells, such as UCBiPS7, was not evaluated.

In summary, the current cardiomyocyte differentiation protocol successfully differentiated UCBiPS7, transgene integrated human cord mononuclear blood cells derived iPSCs, and PCBC16iPS, transgene free human neonatal dermal skin fibroblasts derived iPSCs. hiPSC-CMs had contractility, expressed cardiomyocyte specific transcription factors and myofilament proteins, and exhibited cardiac myocyte-like action potentials. These data, together with the abundance of hiPSC-CMs, demonstrate the potential for cellular therapy for cardiac repair and regeneration.

### Conclusion and Future Work

While the beneficial effects of cellular therapy in hearts with post myocardial infarction LV remodelling (MI) have recently been reported, there could be additional benefit in applying a prefabricated bioartificial cardiac tissue, a “cardiac muscle patch”, over the surface of a myocardial infarct. A “cardiac muscle patch”, formed by entrapping human cardiac myocytes in a PEGylated fibrin 3D porous biomaterial, has recently become possible as the basis for improving cellular therapy for myocardial repair [21]–[23] We have recently established novel 3D porous PEGylated fibrin biomaterial that can covalently bind to growth factors to create an optimal microenvironment for cells to reside [21]–[23] and differentiate. Thus, future experiments will use the PEGylated biomaterial, which also functions as a controlled prolonged release of growth factors to mobilize endogenous cardiac progenitors and to prevent apoptosis [23]–[24]. The objective of the research project is to fabricate cardiac muscle patch using hiPSC derived cardiac cells. Our preliminary data (not shown) also indicate that a PEGylated fibrin 3D porous biomaterial that seeded with hiPSC-CM as well as the hiPSC derived endothelial cell and smooth muscle cells can develop into a contracting cardiac muscle sheet, which beat continuously for many weeks. We will fabricate the contracting human cardiac muscle sheet from hiPSCs of patients with different types of congestive heart failure (CHF). The developed human cardiac muscle sheets will be used to examine mechanisms of LV contractile dysfunction of CHF patients, to test new drugs in treating heart failure, and to be used as a patch surgical therapy for hearts with myocardial infarction.

## Supporting Information

Movie S1 **Contracting sheet of CMs differentiated from the transgene integrated UCBiPS7 on day-1 after initiating contracting**. (Magnification = 25x).
(WMV)

Movie S2 **Contracting sheet of CMs differentiated from the transgene integrated UCBiPS7 on day-5 after initiating contracting.** (Magnification = 25x).
(WMV)

Movie S3 **Contracting sheet of CMs differentiated from the transgene integrated UCBiPS7 day-10 after initiating contracting.** (Magnification = 25x).
(WMV)

Movie S4 **Contracting sheet of CMs differentiated from the transgene-free PCBC16iPS on day-2 after initiating contracting.** (Magnification = 25x).
(WMV)

Movie S5 **Contracting sheet of CMs differentiated from the transgene-free PCBC16iPS on day-6 after initiating contracting.** (Magnification = 25x).
(WMV)

Movie S6 **Contracting sheet of CMs differentiated from the transgene-free PCBC16iPS on and day-11 after initiating contracting.** (Magnification = 25x).
(WMV)

Movie S7 **Intracellular Ca concentration oscillated in hiPSC-CMs.** Images were background subtracted and displayed in a false color scale. Oscillations of Ca concentration in cells in clusters were often synchronized, suggesting that the cells were physiologically coupled.
(WMV)
